# GENESISS 1—Generating Standards for In-Situ Simulation project: a scoping review and conceptual model

**DOI:** 10.1186/s12909-022-03490-9

**Published:** 2022-06-20

**Authors:** Bryn Baxendale, Kerry Evans, Alison Cowley, Louise Bramley, Guilia Miles, Alastair Ross, Eleanore Dring, Joanne Cooper

**Affiliations:** 1 Trent Simulation & Clinical Skills Centre, Nottingham University Hospitals NHS Trust, Nottingham, Notts UK; 2 Institute of Care Excellence, Nottingham University Hospitals NHS Trust, Nottingham, UK; 3 Nottingham University Hospitals NHS Trust, Research and Innovation, Nottingham, UK; 4 Glasgow Dental School, University of Glasgow, Glasgow, UK

**Keywords:** In-situ simulation, Simulation-based education, Clinical training, Simulated practice, Health professions

## Abstract

**Background:**

In-Situ Simulation (ISS) enables teams to rehearse and review practice in the clinical environment to facilitate knowledge transition, reflection and safe learning. There is increasing use of ISS in healthcare organisations for which patient safety and quality improvement are key drivers. However, the effectiveness of ISS interventions has not yet been fully demonstrated and requires further study to maximise impact. Cohesive programmatic implementation is lacking and efforts to standardise ISS terms and concepts, strengthen the evidence base and develop an integrated model of learning is required. The aim of this study was to explore the current evidence, theories and concepts associated with ISS across all areas of healthcare and develop a conceptual model to inform future ISS research and best practice guidance.

**Methods:**

A scoping review was undertaken with stakeholder feedback to develop a conceptual model for ISS. Medline, OpenGrey and Web of Science were searched in September 2018 and updated in December 2020. Data from the included scoping review studies were analysed descriptively and organised into categories based on the different motivations, concepts and theoretical approaches for ISS. Categories and concepts were further refined through accessing stakeholder feedback.

**Results:**

Thirty-eight papers were included in the scoping review. Papers reported the development and evaluation of ISS interventions. Stakeholder groups highlighted situations where ISS could be suitable to improve care and outcomes and identified contextual and practical factors for implementation. A conceptual model of ISS was developed which was organised into four themes: 1. To understand and explore why systematic events occur in complex settings; 2.To design and test new clinical spaces, equipment, information technologies and procedures; 3. To practice and develop capability in individual and team performance; 4. To assess competency in complex clinical settings.

**Conclusions:**

ISS presents a promising approach to improve individual and team capabilities and system performance and address the ‘practice-theory gap’. However, there are limitations associated with ISS such as the impact on the clinical setting and service provision, the reliance of having an open learning culture and availability of relevant expertise. ISS should be introduced with due consideration of the specific objectives and learning needs it is proposed to address. Effectiveness of ISS has not yet been established and further research is required to evaluate and disseminate the findings of ISS interventions.

**Supplementary Information:**

The online version contains supplementary material available at 10.1186/s12909-022-03490-9.

## Background

In healthcare, scenario-based simulation increasingly involves multi-professional teams with the aim of enhancing the application of knowledge, embedding evidence-based practice and improving the performance of the team as a whole [1, 2]. In-situ simulation (ISS) enables teams to practice in the clinical environment where genuine care takes place, and has principally focused on low probability, high consequence events [3]. ISS may offer additional benefits to traditional ‘education-centre’ based simulation, enabling participants to problem solve within their own dynamic setting and facilitating the contextualised implementation of learning into practice [4, 5]. ISS can be a mechanism to explore the interplay between and within micro systems (individual, team and task factors) and macro level phenomena (hospital departments, facilities and systems; [6]), enabling latent safety threat (LST) detection leading to improvements in safety and performance [1, 7]. ISS models are relevant to various healthcare settings as they are flexible to different contexts [8]. There may also be economic benefits associated with ISS when compared to simulation training undertaken in dedicated simulation suites [4, 9]. During the COVID-19 pandemic, ISS interventions have been used to test the ability of healthcare teams to effectively implement use of personal protective equipment (PPE), test infection control guidelines and operational readiness of intensive care units and operating rooms [10–14].

Haji et al. [15] developed a theory-based, iterative, programmatic framework for simulation interventions adapted from the Medical Research Council (MRC) framework for complex interventions [16]. Multiple theories were identified which are applicable to stages of simulation development: learning or instructional design theories for intervention development and modelling; cognitive and behavioural science where participant behaviour change or transfer of knowledge and skills is required; socio-cognitive theories for team based training; and implementation and complexity science to explore integration and contextual factors [15]. Although there is a paucity of conceptual, planning and evaluation frameworks which are solely focused on ISS, components of existing conceptual frameworks for general simulation training and existing literature reporting and evaluating ISS interventions may provide a useful starting point.

The National Simulation Development Project Report (The Higher Education Academy, Association for Simulated Practice in Healthcare (ASPiH) & Health Education England, 2014) identified that the use of ISS is increasing within the United Kingdom (UK) but requires more comprehensive and cohesive strategic and operational support to achieve the potential benefits offered. Efforts to standardise ISS terms and concepts and develop an integrated model of learning is required [2, 4, 17]. Previous reviews have focused on ISS for education and training of healthcare professionals [18], ISS in operating rooms [19], effect on patient outcomes [20] or have included ISS within a broader simulation approach within acute care settings [21], for caesarean section training [22] and obstetric emergency teams [23]. Reviews have highlighted a limited but promising evidence base for ISS and reported high variability of ISS approaches to design, delivery, and evaluation [18–23].

The aim of this study was to explore the current evidence, theories and concepts associated with ISS across all areas of healthcare and develop a conceptual model to inform future ISS research and best practice guidance.

## Methods

This study involved three components:

1. A scoping review of the current literature relating to ISS
2. Consultation with stakeholders from clinical and health education organisations
3. Development of conceptual and logic models of ISS interventions

### Component one: scoping review

We selected a scoping review as they are designed to explore the extent, range, and nature of the emerging evidence [24–26]. A scoping review would enable us to categorise the concepts and theoretical approaches for ISS in healthcare and develop theories about how distinctive mechanisms of ISS (‘natural teams in natural settings’) have the potential for addressing specific learning and clinical needs for the individual, team and various organisation levels. We conducted a scoping review of the published ISS literature following the framework by Levac et al. [27].

The scoping review aimed to address the following questions:

- *What types of ISS healthcare interventions have been evaluated and reported?*
- *What were the reported objectives, design and outcomes reported in ISS studies?*

Studies were included in they: 1) included any type of healthcare professionals and/or healthcare support workers as participants; 2) reported ISS interventions as part or whole of an intervention; 3) were conducted in any healthcare setting including primary or secondary care. The scoping search was limited to OECD countries. Studies which conducted simulation in laboratory, off site or training facilities, were solely focused on pre-registration or undergraduate participants were excluded.

A two-step search strategy was used, the initial search was conducted in September 2018 and updated on 10th December 2020. The search included papers published in the English language from inception to December 2020. Due to time restrictions, we limited the search to three database: Medline, OpenGrey (now archived in the DANS EASY data archive) and Web of Science.

Broad search terms were developed and refined by the study team [27] which included ‘simulation; drills; simulation training; patient simulation’ AND ‘in situ; clinical care; practice; real world; point of care; workplace’ (Additional file 1). Reference lists from all identified studies were reviewed for additional citations to enhance rigour of the approach. Searches and screening were completed by one reviewer (KE). Full text review was completed by KE and members of the study team. Agreement was reached through group discussion. The process of charting the data was conducted by two reviewers (KE, LB) from included papers using a standardised data extraction form to record characteristics of the included studies and the key information relevant to the review question [28].

Data were analysed using a descriptive approach, summarising the data and study characteristics. Data were then organised into categories based around the different motivations, concepts and theoretical approaches for ISS as identified in previous reviews [27, 29]. This was an iterative process completed through discussion with the project team and further refined through the stakeholder engagement.

Guidelines: The scoping review was conducted following the methods described by Levac et al. [27]. The protocol development and scoping review reporting was guided by the Preferred Reporting Items for Systematic reviews and Meta-Analyses extension for Scoping Reviews (PRISMA-ScR) Checklist [29].

### Component two stakeholder engagement

Levac et al. [27] recommend incorporating consultation with stakeholders as a required knowledge translation component of scoping study methodology. We sought expert feedback on the preliminary scoping review findings to build on the evidence and offer a higher level of meaning, content expertise, and perspective to inform the conceptual model [27, 30]. An ISS workshop was held at the Association of Simulated Practice in Healthcare (ASPIH) national conference (2018). As part of the workshop, attendees were presented with the preliminary study findings. A ‘World Café’ method [31, 32] with four × 10 min small group discussions which focused on the ISS categories identified from the preliminary findings. Participants were presented with a series of questions What are the benefits (if any) of ISS over other methods?

- What type of enquiry is more suited to ISS and why?
- What type of ISS design should be used for different types of enquiry?
- How can ISS support psychological safety of participants?

Data from flipcharts, posters and facilitators field notes, were transcribed and summarised.

Attendees from higher education and healthcare provider institutions including multi-professional clinical and managerial staff groups attended the workshop.

Component three: developing a conceptual model of ISS ion healthcare settings.

Data from the scoping search findings, stakeholder discussion and debate were synthesised and developed into a conceptual model of the concepts and theories associated with ISS in healthcare settings to address specific contextual needs. Logic models were then developed to identify short, medium and long-term outcomes that are linked to the key activities of ISS mechanisms [33].

## Results

The results of the three components will now be presented.

### Component one: scoping review

**Fig. 1:**
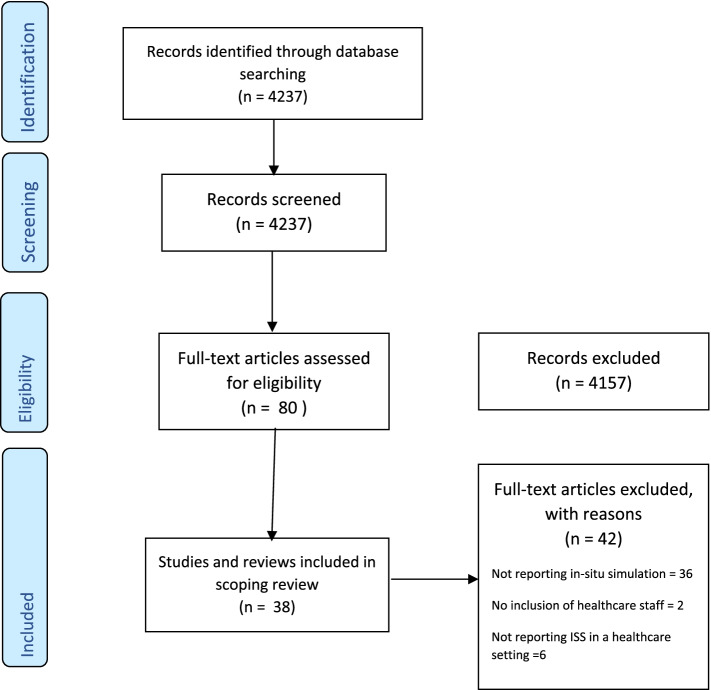
PRISMA diagram – Papers describing ISS interventions in healthcare settings

The search identified 4237 papers which were assessed for eligibility via title and abstract. Eighty papers were retrieved for full text assessment, following discussion and agreement with the review team, 3 reviews and 35 studies were selected for inclusion (Fig. 1, Additional file 2). Papers were from Australia, Canada, Denmark, France, Hong Kong, Israel, Japan, Netherlands, Norway, Sweden, Switzerland, UK and the US and publication dates ranged from 2008 to 2018.

### Systematic reviews of ISS interventions

An umbrella review of simulation-based training for nursing education and practice [34] included one paper focused on ISS [35]. ISS was found to foster improved competencies related to patient safety and collaborative practice such as interdisciplinary communication and teamwork, as well as identifying and correcting actual clinical safety issues. Rosen et al. [18] conducted a systematic review of ISS interventions that included 29 papers which were focused mainly on surgical or maternity care. Most studies were rated as low quality. Approaches to design, delivery, and evaluation of ISS were highly variable across studies. Formal needs analysis was rarely used to develop simulations, there was little evidence of formal training or performance management for facilitators and few programmes reported meaningful evaluations of programme effectiveness. However, a positive impact of ISS on learning and organisational performance was demonstrated in a small number of studies. Owei et al. [19] conducted a systematic review of operating room ISS which included 19 papers, describing its application for a variety of purposes and in a variety of settings, all premised on the potential to offer unique advantages over other types of simulation. One randomized controlled trial (RCT) comparing ISS to off-site simulation found few significant differences. One large-scale outcome study showed improved perinatal outcomes in obstetric care. The authors concluded that although ISS theoretically offers certain advantages over other types of simulation, especially in addressing system-wide or environmental threats, its efficacy has yet to be reliably demonstrated.

### Characteristics of studies reporting ISS interventions

The remaining papers reported ISS interventions conducted in maternity care settings, paediatrics, neonatal, trauma and emergency departments, resuscitation response teams, nursing, mental health and primary care settings. Papers reported various methods for evaluating ISS interventions including: RCTs, surveys, focus groups, cohort studies, observation (pre and post intervention studies) and clinical audit. Papers reported ISS interventions lasting from 15 min to 12 h, describing exercises that were announced and unannounced, and providing single or repetitive ISS sessions. Most papers reported that ISS had been developed by clinical educators and senior clinical staff. Various frameworks for designing, conducting and evaluating ISS were utilised, including systems science models, such as the Systems Engineering Initiative for Patient Safety (SEIPS) model [36], and improvement science models such as the ‘plan, do, study, act’ (PDSA) cycle [37].

Studies aimed to evaluate ISS on various outcomes (Table 1), including:

- Clinical knowledge, technical or procedural skills and response times [4, 5, 38–58]
- Non-technical skills and teamwork behaviours [5, 8, 36, 39, 41, 44, 45, 48–52, 55, 57, 59–63]
- Examining system and process design and detecting LSTs [6, 36, 43, 47, 50–53, 55, 62–65]
- Exploring organisational changes and measuring impact [36, 56, 66–68]
- Participant views and perceptions [45, 57, 61]

**Table 1:** Objectives and outcome measures of the studies in the scoping review

**First author / Year**	**Objectives**	**Participants *Setting***	**ISS length and frequency *Announced / unannounced (if reported)***	**Outcomes: Clinical knowledge, technical or procedural skills and response times**	**Outcomes: Non-technical skills and teamwork behaviours**	**Outcomes:** S**ystem and process design, LST detection**
Amiel 2016 [48]	ISS to evaluate and to train trauma teams	Interprofessional trauma teams *Hospital*	45-min ISS before and after a training intervention	Advanced Trauma Life Support skills, safety	Teamwork, communication	
Barbeito 2015 [36]	Monitor cardiac arrest response process for hazards and defects. Detect opportunities for system optimisation	Interprofessional CPR teams *Hospital locations*	72 ISS cardiac arrest sessions over 3 years *Unannounced*		Teamwork and culture hazards and defects	Environmental, human machine Interface and policy hazards and defects
Ben-Ari 2018 [38]	improve safer practice of ED sedation by paediatricians	Paediatricians *Paediatric ED*	ISS with debrief followed by a second ISS (2–9 weeks later). *Unannounced*	Patient safety task performance related to sedation		
Bender 2011 [6]	TESTPILOT implementation to demonstrate improved system readiness and staff preparedness	Interprofessional teams *New neonatal Intensive Care Unit*	30-min ISS over 4 days			System readiness and identification of LSTs
Brandstorp 2016 [8]	Explore the local learning processes and to improve ISS team training in the primary care emergency teams with a focus on interaction	Primary care Interprofessional teams *Rural Primary Care settings*	Monthly, one-day training sessions		Participants reported understanding of communication and developed local procedures	
Chen 2017 [66]	Assess the readiness of a new department	Interprofessional teams *Hospital facility*	ISS over 4 phases in 3 h *Unannounced*			Identification of process and system issues
Fialkow 2014 [39]	Development, content validation, and implementation of a post-partum haemorrhage (PPH) ISS	Interprofessional teams *Obstetric units*	Two-hour training with a 20-min ISS followed by a debrief *Unannounced*	Participants perceived benefit of the ISS for managing clinical emergencies	Participants perceived benefit of ISS for teamwork learning	
Geis 2011 [52]	Assess the Safety of New Healthcare Teams and New Facilities	Interprofessional teams *Emergency department*	Two 8-h ISS scenarios	Clinical proficiencies	Teamwork behaviours	Identification of LSTs
Gibbs 2018 [53]	Mitigate an Outbreak of Methicillin-Resistant Staphylococcus aureus (MRSA)	Interprofessional teams Neonatal Intensive Care Unit	30-min ISS over 2 weeks	Compliance with hand hygiene, knowledge about infection control. MRSA rates of infection		Diagnose and correct LSTs
Gundrosen 2014 [59]	Asses the feasibility of ISS and assessing non-technical skills	Nurses *Intensive care*	one-hour training followed by ISS assessment		Team competence and non-technical skills	
Härgestam 2016 [49]	Investigate factors associated with the time taken to decide to go to surgery	Interprofessional teams *Emergency departments*	Single ISS with 16 trauma teams	Time taken to decide to go to surgery (seconds)	Closed loop communication	
Herbers 2016 [40]	increase confidence levels and improve nursing performance during emergencies	Nursing staff *Medical and vascular surgical progressive care*	Regular ISS conducted over a 2-year period	Response times and nurses’ confidence		
Jung 2016 [41]	Increase knowledge of how to perform during a disaster, improve skills and communication	Interprofessional teams *Emergency departments*		Knowledge, skills and	Participants’ communication scores	
Kelsey 2016 [4]	Understand how to safely prioritize a difficult care situation and manage workload	Nursing staff *Inpatient medical-surgical nursing unit*	Over 12 h with 3 short ISS embedded *Unannounced*	Identification of additional educational needs. Nurses’ knowledge and comfort		
Knight 2014 [58]	Resuscitation Team Training to improve survival to discharge and code team performance	Interprofessional teams *Children’s hospital*	Monthly ISS over 6 months *Unannounced*	Survival rates, morbidity, team performance		
Kobayashi 2012 [54]	ISS to improve safety of Emergency department procedural sedation (EDPS)	Physicians *Trauma centre*	10 ISS scenarios over 3 months	Skills confidence levels		
Kobayashi 2013 [68]	Determine performance characteristics of a telemetry system	Interprofessional teams *Emergency department*	ISS over three 2-week periods (pre-post intervention)	Simulated arrhythmia detection		System performance
Kurosawa 2014 [42]	Feasibility and effectiveness of ISS Paediatric Advanced Life Support training for recertification	Nurses and respiratory therapists *Paediatric Intensive Care Unit*	Six 30-min ISS over 6 months *Announced*	Clinical performance scores	Behavioural scores	
Lavelle 2017 [5]	Improve knowledge, confidence, and attitudes towards managing medical deterioration	Interprofessional teams *Mental health wards*	Eight half-day sessions (weekly) *Staff aware that training was taking place*	Knowledge, confidence and attitudes managing medical deterioration	Understanding effective communication, self-reflection, team working	
Lutgendorf 2017 [57]	Develop and implement a comprehensive, high fidelity, obstetric simulation	Interprofessional teams *Obstetric unit*	2-day period, with 8 ISS per day	Comfort levels managing obstetric emergencies. Clinical outcomes and response times	Perceived benefit on teamwork and communication	
Marshall 2015 [46]	Evaluate ISS and team training for PPH	Interprofessional teams *Obstetric units (urban and rural communities)*	ISS with debrief and training – repeated 9–12 months later	Response times—recognition of PPH, administer medication, performance of uterine massage		
Medwid 2015 [64]	ISS to identify LSTs, improve layout and workflow, orient staff and decrease stress during the first few weeks of opening	Interprofessional teams *Emergency department*	15 ISS throughout the day			Orientation, identification of LSTs
Miller 2012 [60]	ISS to improve perinatal safety	Interprofessional obstetric and neonatal staff *Six hospitals*	35 ISS events of obstetric emergencies		Teamwork behaviours	
O’Leary 2014 [55]	Identify suboptimal care during simulated scenarios and identify the potential causation factors	Interprofessional teams *Emergency department and operating theatre*	73 ISS over 9 months	Knowledge and clinical skill deficits, drug choice and doses, advanced airway and ventilation, intravenous fluids and recognition of the deteriorating patient	Leadership, communication, planning, situational awareness	Causes of suboptimal care
Patterson 2013 [50]	ISS to promote identification of LSTs and systems issues	Interprofessional teams *Emergency departments*	90 ISS over 1 year. 10-min ISS and 10-min debrief	Perceived values of ISS on learning outcomes Clinical impact	Teamwork scores	Identification of LSTs
Rubio-Gurung 2014 [43]	To determine whether ISS training improved neonatal resuscitation	Interprofessional maternity teams *Maternity units*	4-h training: Two 10-min ISS and debrief	Technical skills	Teamwork score	Hazardous events
Siegel 2015 [44]	Assess emergency department procedural sedation	Senior emergency medicine residents *Emergency department*	2 ISS scenarios	Safety practices	Performance skills	
Sørensen 2014 [45]	Impact and participant perception of ISS	Interprofessional maternity teams *Maternity unit*	ISS drills obstetric emergencies *Unannounced*		Participant perception of ISS: stress, anxiety perceived benefit	
Sørensen 2015 [61]	Effect of ISS versus off-site simulation on knowledge, patient safety attitude, stress, motivation, team performance and organisational impact	Interprofessional maternity teams *Maternity units*	18–26 min ISS followed by a debrief *Announced*	Knowledge scores and patient safety attitude	Stress measurements, motivation and teamworking	
Thelian 2017 [56]	Evaluate the long-term impact of ongoing regular team training on hospital response to deteriorating ward patients	Interprofessional teams *Paediatric hospital*	Weekly team training with ISS lasting 2-h	Patient outcomes and admissions (pre/post intervention)		
Ventre 2014 [63]	Evaluate Operational Readiness of a Children’s Hospital-Based Obstetrics Unit	Interprofessional teams *Obstetric unit*	3 ISS scenarios across several weeks		Interprofessional communication	Identification of operational deficiencies and system issues
Wheeler 2013 [62]	ISS to improve quality and safety	Interprofessional teams *Children’s hospitals*	Regular 10-min ISS and 10-min debrief	Participants’ knowledge	Teamwork behaviours	Identification of LSTs
Yager 2016 [47]	ISS to identify latent inefficiencies and allow rapid intervention testing to improve performance	Interprofessional teams *Paediatric intensive care unit*	12 20-min ISS paediatric emergencies over one year (40 min debrief)	Response times following inefficiencies identified during ISS and implementing improvements		Process inefficiencies
Yajamanyam 2015 [65]	In situ simulation as a quality improvement initiative	Interprofessional teams *Paediatric emergency department and neonatal unit*	29 45-min ISS across the units			Identification of LSTs
Zimmerman 2015 [51]	Development, implementation and impact of an ISS team and resuscitation training program	Interprofessional teams *Children’s hospital units*	Regular monthly ISS and debrief	Learning needs assessment	Communication, leadership	LSTs and system changes

### Component two: stakeholder workshops

The stakeholder group indicated formative rather than summative ISS assessments have the potential to improve learning and encourage Healthcare Professionals (HCPs) to identity their own training needs. Formal teaching and traditional simulation assessment programmes often fail to prepare staff for the ‘real world’ and ISS may help apply learning and reduce the theory-to-practice gap. However there are no robust strategies or non-technical skills standards to assess behaviours, attitudes and communication in changing complex and dynamic settings. It would be very difficult if not impossible to present all participants with equal opportunities to demonstrate their skills during ISS. It was suggested that ISS assessment interventions should:

- aim for realism rather than hyper realism; attempts to exaggerate or exacerbate scenarios should be avoided.
- have clear learning objectives; the object of assessment (i.e. individual, team and/or system) should be defined as each requires a different approach and assessment technique.
- have objectives informed by training needs analyses which form part of an integrated curricula employing numerous learning and assessment approaches. The ‘gaps’ in learning which ISS can address should be clearly identified.
- ensure information including learning resources is standardised and available for staff to access prior to ISS implementation.
- ensure ISS is conducted within supportive learning cultures which have well defined supportive and training packages.
- provide facilitators with specialist training including peer assessment.
- ensure ISS interventions involve creative planning to avoid being continually cancelled in busy departments; consider alternative setting such as staff areas and social spaces.

Stakeholders suggested that ISS has potential to complement traditional investigation approaches but that not all clinical incidents were thought to be appropriate for ISS exploration. Comments provided during the workshop were formed into a series of questions to help HCPs and healthcare educators decide if ISS is an appropriate intervention to aid in the investigation of clinical incidents with a view to foster deeper learning of the factors involved and how these might be mitigated in future (Additional file 3).

Component three: developing a conceptual model of ISS in healthcare settings.

**Fig. 2:**
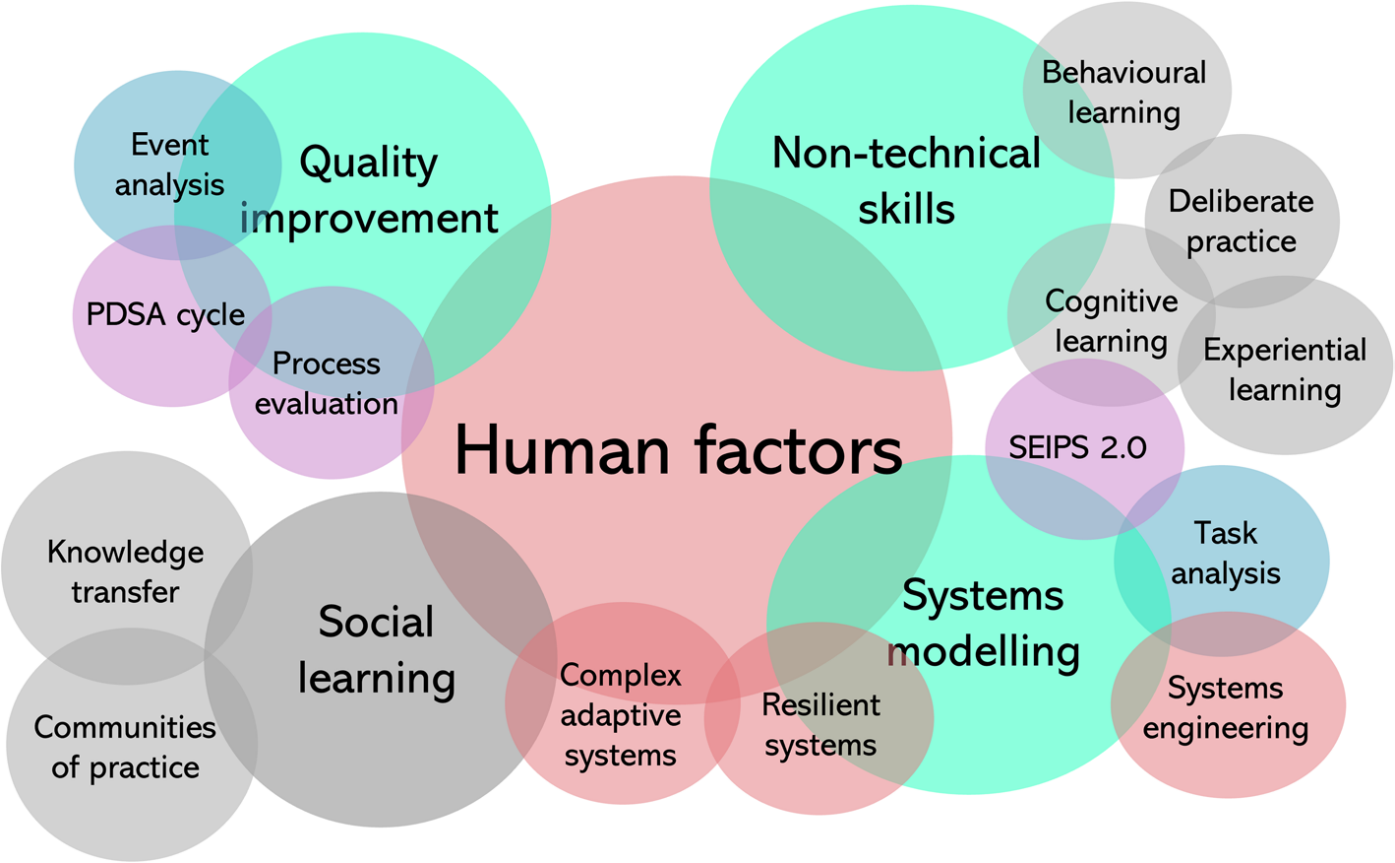
In-situ simulation principles, theories and approaches used in healthcare settings

The scoping review findings and stakeholder feedback identified various principles, theories and approaches for ISS in healthcare settings (Fig. 2).

**Fig. 3:**
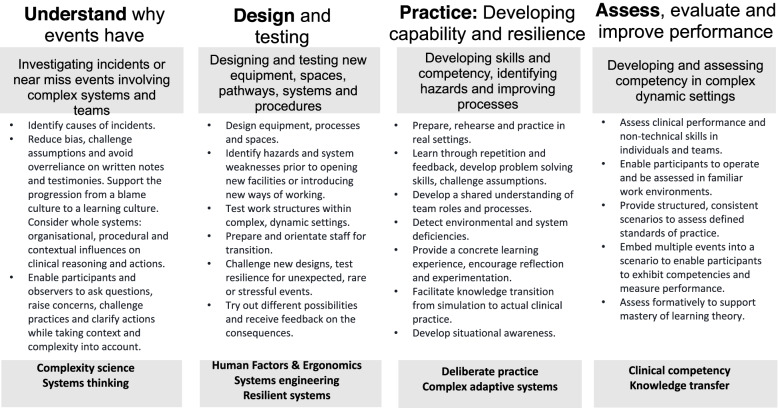
Conceptual model of ISS for healthcare settings

Four distinct concepts were identified (Table 2, Fig. 3):

- ISS to understand why errors have occurred
- ISS to design and testing new equipment, spaces, pathways, systems and procedures
- ISS to practice skills and develop competency
- ISS to assess, evaluate and improve performance

**Table 2:** Functions of in-situ simulation in healthcare

**ISS approach** *Scoping studies*	**Objective**	**Object of enquiry**	**Not suitable for**	**Outcomes**
**Understand** [36, 45, 47, 50, 55, 58, 61, 62, 65]	Explore why events occur in complex settings; enable learning from critical or significant untoward incidents	Healthcare systems, processes, environments, pathways and procedures	Individual assessment of performance; incidents considered to have obvious single causes	Identifying and addressing system weaknesses (“latent factors”) to improve quality and safety
**Design **[6, 52, 63, 64, 66, 68]	Design and testing of new work systems and processes including clinical spaces, equipment, information technologies, procedures and pathways	Clinical environments; pathways; complex procedures; equipment performance in dynamic settings; organisational readiness	Behavioural change: the focus is on improving the work system, environment or equipment to meet staff or patient needs	Proactive identification of anticipated unintended consequences; improve efficiency by addressing issues including flow, usability, accessibility and familiarity of space / equipment / procedures
**Prepare **[4, 5, 8, 38–41, 43, 46, 48–51, 53, 56, 57, 60, 62]	Practice and develop capability of individuals and team performance	Individual and team performance; non-technical skills and team behaviours; adaptability in performance and resilience during high consequence events	Scenarios which do not involve clinical teams or dynamic settings	Understand the roles and responsibilities of team members and the impact of the clinical environment; identify latent threats and vulnerabilities; improve systems, processes and identify learning needs
**Assess **[4, 38, 42–44, 51, 53, 56, 59]	Assess competency in complex clinical settings	Formative or summative assessment of individual and team performance	Organisations which do not foster a ‘just culture and safe learning environment’	Reduce the theory and practice gap; ensure preparation of staff for real world experiences and identify further learning needs; offer assurances to patients, public, employers and regulators

## Discussion

**Fig. 4:**
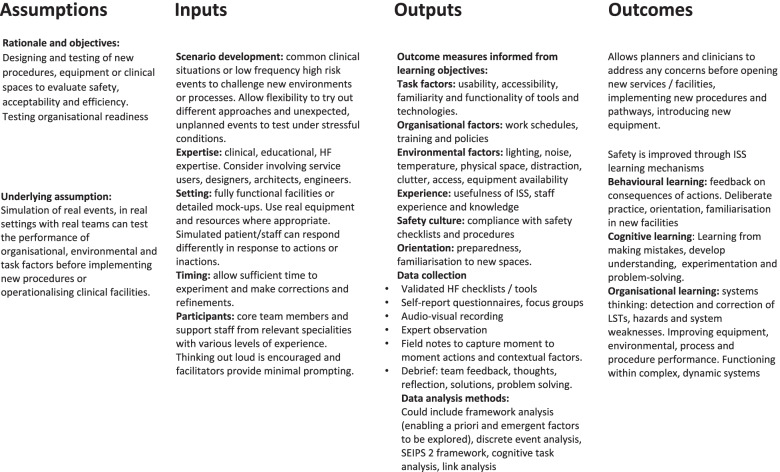
Logic model: *Design* ISS

**Fig. 5:**
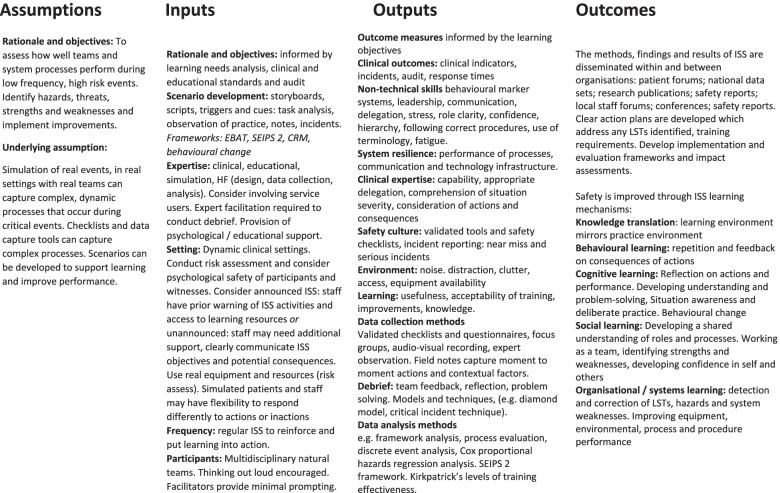
Logic model: *Practice* ISS

The four concepts of ISS are presented alongside logic models (Figs. 4 and 5) with discussion of how each ISS approach may be developed to address specific contextual needs.

### In-situ simulation: understand why events have occurred

Learning from adverse incidents should move beyond attributing cause wholly to human failings and more towards investigating the role of the system in which humans operate [69–71]. Root cause analysis (RCA) investigations promote a systematic approach to investigating serious incidents, although RCA may impede organisational learning as it can restrict explanation to single causes and is often over reliant on individual testimonies or medical notes [72]. Alternative ways of capturing less reductive incident ‘stories’ can encourage reflection and wider organisational learning, considering the interaction between a range of systemic factors which contribute to incidents. ISS may provide a useful mechanism for such inquiry, assisting clinicians to discover potential interacting components and identify additional LSTs [65, 73–75].

As part of a wider approach which aims to encourage healthcare organisations to progress from a ‘blame culture’ to a just, learning culture [71], embedding simulation activities underpinned by Human Factors principles can help to focus on the organisational, procedural and contextual influences on clinical reasoning and actions. Participants of ISS interventions should be encouraged to observe, reflect, ask questions, raise concerns, challenge practices and clarify actions while taking context and complexity into account.

Patient involvement in the design and delivery of simulation training enables the patients’ experience to be expressed and considered. Many ISS scenarios have been developed to reflect ‘real life’ risks drawn from patterns in care, sentinel events, or concern from advocacy groups. However it maybe be possible to work in collaboration families involved in a serious incident to help facilitate learning opportunities and disseminate the findings [73]. This would involve careful attention to local sensitivity; where harm has occurred this of course may be distressing for staff, patients and families involved (particularly where organisations have a culture of appropriating blame and censure). The acceptability of co-designed ‘reconstruction’ via ISS scenarios from care histories should always assess the potential effect on patients and staff involved.

### In-situ simulation: design and testing

Tools of safety and complexity science such as Discrete Event Simulation [76], Cognitive Task Analysis [77] and general system thinking models have been used to design ISS interventions to enhance patient flow, improve the design of clinical spaces, and identify LSTs within new emergency and obstetric departments. For example, Bender [6] scripted commonly encountered clinical scenarios to explore the functioning of a new perinatal facility. Scenarios challenged participants to test the new facilities under stressful conditions, focusing on identifying LSTs and making improvements which were more readily adopted into practice as they were driven the clinical teams. Medwid et al. [64] used ISS to test a new emergency department; numerous LSTs were identified and addressed prior to the department opening. ISS facilitated deliberate practice within the new space and helped orientate staff to the new facility. ISS has also been reported to assist the development and testing of new clinical services and procedures for cardiac services [66] and stroke thrombolysis pathways [78]. The MHRA guidance for medical devices [79] highlights that usability testing with representative users in a simulated environment or the actual environment of use can help identify which device features people find easy to use and which cause problems, thus determining whether the device is susceptible to user errors that could cause harm.

Design-driven improvement is a core Human Factors principle which can benefit healthcare organisations [80, 81]. The performance of a process is the emergent property of the whole interacting system which is complex, dynamic and situation specific [82, 83]. The ability to experiment and see what occurs through interactions, attunement and disturbances enables participants to question how things might be done differently, try out various options and consider possible unintended outcomes [84]. ISS can be designed to test the synergy or dissonance between micro and macro factors: task factors, organisational factors, internal environments and external environments [81]. The logic model proposed in Fig. 4 has been developed with reference to the current literature and approaches to ISS design and testing interventions. The model highlights the mechanisms of ISS which would be potentially amenable to standardised approach.

### In-situ simulation: practice, developing capability and resilience

Organisational resilience is focused on understanding how healthcare organisations can deliver standardised, replicable and predictable services while embracing inherent variations, disruptions and unexpected events [85]. This involves designing, testing and improving the organisational systems that support rapid and adaptive responses to emergency situations. Deficiencies and LSTs in current systems can be identified and addressed through ISS activities as they enable a systematic examination to provide a realistic picture of work, contextualised in real time and place [7, 55, 62, 86]. ISS scenarios can also support HCPs to develop non-technical skills: task management; teamwork; situation awareness; problem-solving; and decision-making, while testing and probing real-world organisational systems [4, 43, 85, 87–89]. ISS to assist teams prepare, rehearse and practice for low frequency, high impact events was the most reported ISS activity included in the review and was often underpinned by a combination of established learning theories (Fig. 5).

#### Behavioural learning

Skills are developed through repetition. Learning and behaviour change occurs through feedback from the simulation activity, interaction between the task, environment, and the team. ISS provides opportunity for teams to identify solutions and take action to introduce and reinforce changes [90]. Video playback can support behavioural learning alongside a discussion of strategies, alternative approaches, personal experiences and emotional aspects. Teams and individuals can be supported to identify gaps in knowledge, challenge current procedures and identify and address system weaknesses.

#### Cognitive learning

Preconceptions are explored, and new or unexpected events are presented via the simulation activity to challenge precognitions [91]. Questioning and reflection help participants acquire deeper understanding, develop problem solving skills and new insights [92]. ISS enables learning from making mistakes in a way that would be inconceivable with actual patients; ‘thinking aloud’ helps participants reflect on why they took a particular course of action and present an opportunity to correct their actions [84, 93, 94]. ISS activities should provide a concrete experience, debrief with reflexive observation and conceptualisation, ideally followed by a second scenario for active experimentation [95]. Knowledge transfer is optimal when the learning environment matches the environment in which it will be applied [93]. ISS can help develop and maintain situational awareness [96], for example, awareness of vital signs, medication requirements, actions of other team members and equipment function. Comprehension of a current situation based on these elements, understanding their significance within the environment, and forming a holistic picture helps anticipate future actions and consequences [97].

#### Social learning

Learning through observing others and developing a shared understanding of roles and processes within a team which result in the desired outcomes. ISS enables interdisciplinary team practice while maintaining interaction with the environmental and system factors present during actual patient care events [39]. Individuals and teams explore the social processes involved in building shared expectations, establishing patterns of collective working and building trust between multidisciplinary teams [85]. An open and safe context encourages professionals from different backgrounds to acknowledge their strengths and address their weaknesses in a respectful and trustful manner [8, 85].

### In-situ simulation: assess, evaluate and improve performance

Assessing competency through simulation is well established in the military, nuclear and aviation industries and is used in healthcare to assess clinical competency via objective structured clinical examinations (OSCE). Brunette and Thibodeau-Jarry [93] suggest that simulation, through the application of mastery learning theory can be used to formatively assess competency in clinical environments. Learners are required to achieve a level of mastery in particular essential skills and knowledge before progressing onto new or more advanced practice. Learners progress at their own pace and are provided with opportunities for deliberate practice via simulation. Again, ISS enables participants to operate and be assessed in a familiar work environment [59].

Miller’s pyramid for assessing clinical competence distinguishes between four different levels of competence: knows; knows how; shows how; and does. The level ‘does’ is described as the most accurate way to assess competence in actual clinical practice [98]. However, assessment in actual clinical practice has the potential to distress patients and can be problematic in terms of controlling variables such as task difficulty [59]. Sørensen et al. [99] stress the difference between simulation-based training and simulation-based assessment (SBA). In SBA, participants need to be well informed about the proposed activity and know what will be expected of them [87]. Careful attention needs to be paid to creating a safe learning environment. Validated metrics and standards need to be developed for individuals and teams. Strategies to assess non-technical skills during ISS have been developed for medical students, obstetrics and anaesthesiology and could be refined and adopted for other clinical specialties [100–102].

Griswold et al. [103] identify that for clinical procedures with clear chains of action and well-defined processes and standards, summative assessment via ISS is much simpler than in more “dynamic, multifactorial practices in which cognitive, procedural, and communication skills are simultaneously applied in a team environment” (Griswold et al. 2018, page 170). Measurement methods for assessing competencies involved in complex care processes are less well-defined, and further complicated when individual performance needs to be isolated from the wider team. Concepts such as ‘effective communication’ are subject to interpretation, and clinical outcomes may be attributed to concepts such as teamwork and coordination in addition to individual clinical skills and knowledge [103]. Criterion standards and benchmarks of quality performance need to be defined to reliably and accurately capture the individual performance which is linked to relevant outcomes. Strategies to define and control for difference in skill mix, staffing and capacity pressures are also required.

Simulation for training has been reported as effective when instructional features, such as the event-based approach to training (EBAT) are embedded within the simulation [104]. EBAT has been used in aviation and military environments and relies on the “a priori” embedding of multiple events into the scenario at different time intervals. These events are designed to enable participants to exhibit competencies and measure performance. Scenario development can be facilitated by performing a Cognitive Task Analysis to identify the cues expected to be used to perform complex tasks [104]. Data collection should capture ‘moment-to moment’ actions and behaviours to identify how performance can be improved.

### Limitations

The scoping review sought to identify and categorise ISS approaches within a wide range of healthcare settings, however only three databases were searched. Selection and screening were completed by a single reviewer (KE) and the review protocol was not registered. The included studies were conducted across thirteen countries and the cultural context in which ISS was conducted was not explored within the review. Stakeholder feedback which were used to further inform the development of the conceptual model may not represent the whole picture of the concepts and mechanisms of ISS being conducted in healthcare and health education settings. However, we consider that the scoping review and conceptual model have highlighted key characteristics related to ISS and thus provided a useful starting point to develop more specific questions to addressed by a systematic review or primary research.

## Conclusions

This paper provides an overview of the application of the use of ISS in healthcare settings. The recent literature highlights the heterogeneity in ISS objectives and the complex delivery landscape which has resulted in a lack of an integrated ISS approach across healthcare organisations. Most reported ISS interventions provide little evidence of formal development processes and lack validated outcome measures. ISS in healthcare is often underpinned by Human Factors (HF) principles which overlap and synergise with other approaches, methods and theories including non-technical skills development, quality improvement (QI) methods, and systems modelling. Many existing models and frameworks exist within a wide ISS curriculum as part of a general approach to simulation training. This can result in a lack of clear guidance to inform ISS designs. A conceptual model has been provided to inform discussion and debate about the objectives, feasibility and usefulness of ISS interventions to guide clinicians and educators. We have set out the learning mechanisms intrinsic within ISS and suggest the context in which these mechanisms can be actualised. We have highlighted the potential for ISS to improve the design of clinical spaces and equipment, develop team performance and healthcare systems resilience, and support clinical investigation and competency assessment. An ISS approach presents distinctive advantages to explore and improve clinical team and organisational functioning. In addition, the ability to address the practice-theory gap makes ISS an attractive approach for educators, managers and policymakers as part of quality and safety improvement strategies. However there are limitations associated with ISS, such as the impact on the clinical setting, the provision of an open learning culture and availability of relevant expertise. We strongly recommend that ISS is not introduced without due consideration of the specific objectives and learning needs it is proposed to address. Effectiveness of ISS has not yet been established and further research is required to assess the specific effect of particular ISS designs on clinical outcomes, learning outcomes, team performance, non-technical skill development, acceptability and perceived benefit. We encourage researchers, clinicians and educators to work collaboratively to rigorously design, develop, evaluate ISS interventions and disseminate the findings to further inform the evidence base. We recommend future systematic reviews are conducted to assess ISS intervention effectiveness to guide clinicians, researchers and educators to develop effective ISS interventions and provide useful guidance as they continue to address various clinical concerns by ISS interventions in dynamic settings.

## Supplementary Information

**Additional file 1.**

**Additional file 2.**

**Additional file 3.**

## Data Availability

All data generated or analysed during this study are included in this published article [and its supplementary information files].
