# Supplementary material for: GENESISS 1—Generating Standards for In-Situ Simulation project: a scoping review and conceptual model
Source: BMC Med Educ. 2022 Jun 20;22:479. doi: 10.1186/s12909-022-03490-9 (PMC9208746; doi:10.1186/s12909-022-03490-9)
Supplement: Supplementary file 1 — Additional file 1. [file 12909_2022_3490_MOESM1_ESM.docx]

GENESISS 1 - Generating Standards for In-Situ Simulation project: a scoping review and conceptual model

MEDLINE scoping search strategy

1. HIGH FIDELITY SIMULATION/or PATIENT SIMULATION/ or SIMULATION TRAINING
2. simulat*.mp
3. drills.mp
4. in situ.mp
5. in practice.mp
6. real world.mp
7. point of care.mo or Point-of-Care Systems/
8. WORKPLACE/ or workplace.mp
9. 1 or 2 or 3
10. 4 or 5 or 6 or 7 or 8
11. 9 and 10
12. limit 11 to (English language and humans)
