# Supplementary material for: GENESISS 1—Generating Standards for In-Situ Simulation project: a scoping review and conceptual model
Source: BMC Med Educ. 2022 Jun 20;22:479. doi: 10.1186/s12909-022-03490-9 (PMC9208746; doi:10.1186/s12909-022-03490-9)
Supplement: Supplementary file 2 — Additional file 2. [file 12909_2022_3490_MOESM2_ESM.docx]

**Additional file 2: Studies and reports included in the ISS scoping search**

| **Fist author Year** | Study aims | Summary of the Authors’ findings |
| --- | --- | --- |
| **Amiel 2016**  **[48]** | Evaluate a 1-day instructional intervention using ISS to improve the trauma management capabilities of the trainees. | The mobile ISS -based training demonstrated efficacy both as an assessment tool for trauma teams’ function and an educational intervention when coupled with invitro simulation-based training, resulting in a significant improvement of the teams’ function in various aspects of treatment. |
| **Barbeito 2015 [36]** | ISS to continuously monitor the cardiac arrest response process for hazards and defects and to detect opportunities for system optimization | Used the Systems Engineering Initiative for Patient Safety (SEIPS) model to understand the structure, processes, and outcomes related to the hospital's emergency response system. Multidisciplinary solutions were crafted for each of the hazards detected, and the simulation program was used to iteratively test the redesigned processes before implementation in real clinical settings. |
| **Ben-Ari 2018 [38]** | ISS sedation training (SST) of paediatricians to improve the performance of tasks related to patient safety during sedation in the Emergency Department (ED). | Thirty-two sedations were performed, 16 before and 16 after SST. Sedation-Performance-Score improved from a median of 4 (IQR 2-5) to 6 (IQR 4-7) following SST (p < 0.0009, median difference 2, 95% CI 1-3). SST was associated with improved performance in four SPS components. The findings suggest that sedation simulation training of paediatricians improves several tasks related to patient safety during sedation |
| **Bender 2011 [6]** | ISS for Systems Testing in Newly Constructed Perinatal Facilities | Orchestrating immersive, realistic ISS minimizes surprises at transition and enhances patient satisfaction. Such endeavours substantially elevate the perception of the value of simulation within an institution. |
| **Brandstorp 2016 [8]** | To explore the local learning processes and to improve IS team training in the primary care emergency teams with a focus on interaction. | In situ team training was experienced as challenging, engaging, and enabling. The participants expanded the types of training sites, themes and the structures for participation, improved their understanding of communication and developed local procedures. Challenging, monthly in situ team trainings organised by local health personnel facilitate many types of learning. The flexible training model provides space for the participants’ own sense of responsibility and priorities. |
| **Chen 2017 [65]** | ISS for pre-implementation testing of clinical service in a regional hospital in Hong Kong. | Overall, 17 vital process and system issues were identified during the simulation as potential clinical concerns. They included difficult patient positioning, draping pattern, unsatisfactory equipment setup, inadequate critical surgical instruments, blood products logistics, and inadequate nursing support during crisis. ISS provides an innovative method to identify critical deficiencies and unexpected issues before implementation of a new clinical service. Life-threatening and serious practical issues can be identified and corrected before formal service commences. |
| **Fialkow 2014 [39]** | Describe the development, content validation, and in situ implementation of a standardized patient based, interdisciplinary PPH scenario. | Participants indicated that the simulation was effective, and they would like more frequent sessions with different clinical emergencies. Participants commonly cited teamwork issues (e.g., role clarity, resource management, and communication issues) as important learning points. Overall, requirements for simulation resources and participant time were the greatest obstacles. Reserving the operating room be for training was a significant issue, the inclusion of this component to the simulation was particularly valuable because it raised multiple teamwork and communication issues. |
| **Geis 2011 [52]** | Simulation to Assess the Safety of New Healthcare Teams and New Facilities. | Twenty-four simulations were performed over 3 months before the hospital opening. Laboratory debriefing identified the need to modify provider responsibilities. National Aeronautics and Space Administration-Task Load Index scores and debriefings demonstrated that the medication nurse had the greatest workload during resuscitations. Modifying medication delivery was deemed critical. Lower Mayo High Performance Team Scale scores, implying less teamwork, were noted during in situ simulations. In situ sessions identified 37 LSTs involving equipment, personnel, and resources. |
| **Gibbs 2018 [53]** | To describe the successful implementation of an ISS program to diagnose and correct LSTs in a level 4 NICU to mitigate a MRSA. | Before the simulation intervention, there were 18 patients colonized or infected with a single MRSA clone; after the intervention, there were no new episodes of colonization or infection. An in situ, simulation-based intervention can counter threats to patient safety related to workflow and lapses in infection control practices and improve patient outcomes |
| **Gundrosen 2014 [64]** | The feasibility of using an ISS model to explore team competence in the ICU, and on using parts of the Anaesthetists’ Non-Technical Skills (ANTS) taxonomy for assessing Non-Technical Skills in nursing teams. | Due to high activity in the ICU, 54 nurses completed the ISS. Assessments of the video recordings revealed moderate agreement between the two raters. Observations revealed issues deviating from expected standards of competence.  Conclusion: ISS may be feasible for assessing competence in ICUs. The ANTS appears to be a promising foundation for developing a team assessment tool for ICUs. |
| **Hargestam 2016 [49]** | The association between the time taken to decide to go to surgery and gender, ethnicity, years in profession, trauma team training, closed-loop communication and leadership styles during trauma team training. | Closed-loop communication initiated by the team leader increased the chance of a decision to go to surgery (HR: 3.88; CI 1.02 to 14.69). Only 8 of the 16 teams made the decision to go to surgery within the timeframe of the trauma team training. Conversely, callouts and closed-loop communication initiated by the team members significantly decreased the chance of a decision to go to surgery, (HR: 0.82; CI 0.71 to 0.96, and HR: 0.23; CI 0.08 to 0.71, respectively).  Conclusions: Closed-loop communication initiated by the leader appears to be beneficial for teamwork. In contrast, a high number of callouts and closed-loop communication initiated by team members might lead to a communication overload. |
| **Herbers 2016 [40]** | To increase confidence levels and improve nursing performance during medical emergencies via ISS. | Nursing staff response times for calling for help improved 12%, time elapsed before initiating compressions improved 52%, and time to initial defibrillation improved 37%. Additionally, staff showed an increase in perceived confidence levels. Staff reported their appreciation of the opportunity for hands-on practice with the equipment, reinforcing their knowledge and refining their medical emergency skills. |
| **Jung 2016 [41]** | Examine whether ISS will increase health care providers' knowledge of how to perform during a disaster, improve competency in skills related to those actions, and to improve communication | Results of the project demonstrate that ISS can improve knowledge and communication during a disaster situation. |
| **Kelsey 2016 [4]** | ISS to help nurses understand how to safely prioritize a difficult care situation while managing their entire workload during a full shift. | Nurse educators were able to determine nurses did not consistently recognize subtle signs of clinical deterioration in a patient with a spinal cord injury. Nursing staff reported an increase in knowledge and comfort with spinal cord injury and activation of rapid response at the end of the simulation. Clinical decision-making appears to be less effective when subtle patient-assessment findings are collected and interpreted over time in the presence of a full patient assignment being cared for by a variety of caregivers. |
| **Knight 2014 [68]** | To assess the impact of service improvements implemented because of  latent threats (LTs) detected during ISS | An in-situ simulation program can identify important LTs which traditional reporting systems miss. Subsequent improvements in workplace systems and resources can improve efficiency and remove error traps. |
| **Kobayashi 2012 [81]** | To (1) determine the baseline performance of an Emergency Department (ED) telemetry system implementation and (2) improve system performance | Experimental investigations helped reveal and mitigate weaknesses in an ED clinical telemetry system implementation. In situ simulation and HFE methodologies can facilitate the assessment and abatement of patient safety hazards in healthcare environments. |
| **Kobayashi 2012 [81]** | Emergency department procedural sedation (EDPS) is becoming widespread. Simulation may enhance patient safety through evidence-based training, effective assessment, and research of EDPS operators in pertinent knowledge, skills, processes, and teamwork. | In situ simulation is a useful and relevant means to investigate EDPS patient safety. Pilot sessions have cleared the way for further experimental safety intervention research and development with the simulation-based methodology. |
| **Kurosawa 2014 [82]** | Evaluate ISS for Paediatric Advanced Life Support (RCT) | Clinical Performance Tool score was similar at baseline in both groups and improved after Paediatric Advanced Life Support reconstructed (pre, 16.3 ± 4.1 vs post, 22.4 ± 3.9; p < 0.001), but not after standard Paediatric Advanced Life Support (pre, 14.3 ± 4.7 vs post, 14.9 ± 4.4; p =0.59). Improvement of Clinical Performance Tool was significantly higher in Paediatric Advanced Life Support reconstructed compared with standard Paediatric Advanced Life Support (p = 0.006). Behavioural Assessment Tool improved in both groups: Paediatric Advanced Life Support reconstructed (pre, 33.3 ± 4.5 vs post, 35.9 ± 5.0; p = 0.008) and standard Paediatric Advanced Life Support (pre, 30.5 ± 4.7 vs post, 33.6 ± 4.9; p = 0.02), with no significant difference of improvement between both groups (p = 0.49). |
| **Lavelle 2017 [5]** | To evaluate an interprofessional, ISS intervention for managing medical deterioration in mental health settings. | Participants showed significant improvement in knowledge (p < 0.001), confidence (p < 0.001), and attitudes towards (p < 0.02) managing medical deterioration. Incident reporting increased by 33% following training (7 months post training). Participants reported improved confidence in managing medical deterioration, better understanding of effective communication, improved self-reflection’ team working, and increased sense of responsibility for patients' physical health. |
| **Lutgendorf 2017 [83]** | To assess participant comfort with managing obstetric haemorrhage following our multidisciplinary ISS exercise. | Participants reported a higher comfort level in managing obstetric emergencies after simulation training compared to before training. For managing hypertensive emergencies, the post-training mean score was 4.14 compared to a pretraining mean score of 3.88 (p = 0.01, 95% confidence interval [CI] = 0.06-0.47). For shoulder dystocia, the post-training mean score was 4.29 compared to a pretraining mean score of 3.66 (p = 0.001, 95% CI = 0.41-0.88). For PPH, the post-training mean score was 4.35 compared to pretraining mean score of 3.86 (p = 0.001, 95% CI = 0.36-0.63). Multidisciplinary ISS exercise improves self-reported comfort with managing obstetric emergencies and is a safe and effective way to practice skills and improve systems processes in the health care setting. |
| **Marshall 2015 [84]** | To evaluate ISS and team training for PPH among experienced clinical teams in non-academic hospitals in urban and rural communities. | Team training significantly improved response times in the management of PPH, including the recognition of PPH, time to administer first medication, performance of uterine massage and time to administer second medication.  Simulation and team training significantly improved PPH response times among clinically experienced community labour and delivery teams. |
| **Medwid 2015 [38]** | ISS to investigate latent safety threats prior to opening a new emergency department. | ISS performed prior to the opening of a new emergency department identified 35 modifiable latent safety threats. Simulations were an effective way to orient staff to the new space and seemed to decrease the level of discouragement and irritation of healthcare workers during the first few weeks of the emergency department’s opening. |
| **Miller 2012 [85]** | Evaluate ISS in the emergency department (ED) to improve teamwork and communication measured in the clinical setting. | This study shows that an ISS program can be implemented with participation from all members of a multidisciplinary trauma team in the ED of a Level I trauma centre. While teamwork and communication in the clinical setting were improved during the ISTS program, this effect was not sustained after ISS training sessions were stopped. |
| **O’Leary 2014 [48]** | To identify suboptimal care during standardised simulated scenarios and to identify the potential causation factors. | 194 incidents of suboptimal care were observed and attributed to 325 causation factors. There were 76 knowledge deficits, 39 clinical skill deficits, 36 leadership problems, 84 communication failures, 20 poor resource utilisations, 23 preparation and planning failures and 47 incidents of a loss of situational awareness. During standardised paediatric simulations multiple incidents of suboptimal care have been identified and multiple causation factors attributed to these. Educators should use this information to adapt current training programs to encompass these factors |
| **Patterson 2013 [86]** | Implement and demonstrate  feasibility of ISS to identify LSTs at a higher rate than lab-based training and reinforce teamwork training in a paediatric emergency department (ED). | 218 healthcare providers responded to 90 ISS conducted over 1 year. A total of 73 LSTs were identified; a rate of one every 1.2 simulations performed. In situ simulations were cancelled at a rate of 28% initially, but the cancellation rate decreased as training matured. Examples of threats identified include malfunctioning equipment and knowledge gaps concerning role responsibilities. 78% of participants rated the simulations as extremely valuable or valuable, while only 5% rated the simulation as having little or no value. Of those responding to a post simulation survey, 77% reported little or no clinical impact. Video recordings did not indicate changes in nontechnical skills during this time.  ISS is a practical method for the detection of LSTs and to reinforce team training behaviours. Embedding ISS as a routine expectation positively affected operations and the safety climate in a high-risk clinical setting |
| **Rubio-Gurung 2014 [51]** | To determine whether ISS improved neonatal resuscitation performed by the staff at maternities. Maternity staff | After intervention, the median technical score was significantly higher for scenarios 1 and 2 for the IG compared with the CG (P = .01 and 0.004, respectively), the median team score was significantly higher (P < .001) for both scenarios. In the IG the frequency of achieving a heart rate >90 per minute at 3 minutes improved significantly (P = .003), and the number of hazardous events decreased significantly (P < .001). ISS with multidisciplinary teams can effectively improve technical skills and teamwork in neonatal resuscitation. |
| **Siegel 2015 [87]** | To delineate and experimentally assess EDPS performance and safety practices of senior-level emergency medicine residents through ISS. | Study simulations delineated EDPS and assessed safety behaviours in senior emergency medicine residents, who exhibited the requisite medical knowledge base and procedural skill set but lacked some nontechnical skills that pertain to emergency department microsystem functions and patient safety. The experimental system exhibited limited impact only on in-simulation time-out compliance. |
| **Sørensen 2014 [88]** | To describe how unannounced ISS was perceived by HCPs before and after its implementation, and to describe the organisational impact of ISS. | Questionnaires before implementation revealed that 137/196 (70%) of staff agreed that ISS was a good idea and 52/199 (26%) thought it likely to be stressful and unpleasant. Questionnaires after implementation showed significantly more staff members, 135/153 (89%), thought ISS was a good idea. A significantly higher amount of staff members 50/153 (33%) found it to be stressful and unpleasant, and among midwives, 15/59 (25%) were anxious about ISS, whereas none of the obstetricians reported this. Information obtained through debriefing sessions generated learning points and information required for organisational changes. |
| **Sørensen 2015 [89]** | To investigate the effect of ISS versus off-site simulation (OSS) on knowledge, patient safety attitude, stress, motivation, perceptions of simulation, team performance and organisational impact. | No differences between the two groups were found for the multiple-choice question test, patient safety attitude, stress measurements, motivation or the evaluation of the simulations. The participants in the ISS group scored the authenticity of the simulation significantly higher than did the OSS group. Expert video assessment of team performance showed no differences between the ISS versus the OSS group. The ISS group provided more ideas and suggestions for changes at the organisational level |
| **Theilen 2017 [90]** | To evaluate the long-term impact of ongoing regular ISS team training (pMET) on hospital response to deteriorating ward patients, patient outcome and financial implications. | Deteriorating patients were recognised more promptly (before/1 year after/3years after pMET; median time 4/1.5/0.5 h, p < 0.001), more often reviewed by consultants (45%/76%/81%, p < 0.001) and more rapidly escalated to PICU (median time 10.5/5/3.5 h, p = 0.02). There was a significant reduction in associated PICU admissions (56/51/32, p = 0.02) and PICU bed days (527/336/193, p < 0.001). The total annual cost of training (£74,250) was more than offset by savings from reduced PICU bed days (£801,600 per annum). Introduction of pMET coincided with significantly reduced hospital mortality . |
| **Ventre 2014 [91]** | Using ISS to Evaluate Operational Readiness of a Children’s Hospital-Based Obstetrics Unit. | ISS identified multiple operational deficiencies on the OB unit, allowing us to take corrective action before its opening. This project may guide other children’s hospitals regarding care processes likely to require significant focus and possible modification to accommodate an OB service. |
| **Wheeler 2013 [49]** | Describe ISS in a major children’s medical centre. | We identified 134 latent safety threats and knowledge gaps during ISS, which we categorised as medication, equipment, and/or resource/system threats. Identification of these errors resulted in modification of systems to reduce the risk of error. ISS also provided a method to reinforce teamwork behaviours, such as the use of assertive statements, role clarity, performance of frequent updating, development of a shared mental model, performance of independent double checks of high-risk medicines, and overcoming authority gradients between team members. Participants stated that  the training programme was effective and did not disrupt patient care. ISS can identify latent safety threats, identify knowledge gaps, and reinforce teamwork behaviours when used as part of an organisation-wide safety programme. |
| **Yajamanyam 2015 [35]** | Critical incident analysis of an adverse event that happened in the paediatric accident and ED (A&E) at North Middlesex Hospital identified a latent safety threat (LST) as a potential contributing factor. | Twenty-one sessions conducted in paediatric A&E and eight on NNU. Simulation exercise lasted approximately 45 min with the clinical scenario conducted for 10–12 min. Following each individual session, the lead facilitator maintained a log of LSTs identified. This QI project was successful in detecting LSTs, thus improving acute patient care. Prior communication and support from all relevant departments in the trust, availability of appropriate expertise and close links with the clinical governance system of the trust were vital to our success. |
| **Yager 2016 [92]** | Developing an ISS paediatric emergency response program to identify latent inefficiencies and allow rapid intervention testing to improve performance before implementation at an institutional level. | The initial simulated event allowed identification of inefficiencies including delayed provider response, delayed initiation of CPR, and delayed vascular access. These gaps were linked to process issues including unreliable code pager activation, slow elevator response, and lack of responder familiarity with layout and contents of code cart. From first to last simulation with multiple simulated process improvements, code response time for secondary providers coming from the second hospital decreased from 29 to 7 min, time to CPR initiation decreased from 90 to 15 s, and vascular access obtainment decreased from 15 to 3 min. |
| **Zimmerman 2015 [50]** | To describe and evaluate the implementation of an inter-professional ISS team and resuscitation training in a teaching hospital with a programmatic  approach. | Designed and implemented a team and resuscitation training program according to Kern’s six steps  approach for curriculum development. The general and specific needs assessments clearly identified the problems, revealed specific training needs and assisted with stakeholder engagement. Ninety-five interdisciplinary staff members of the Children’s Hospital participated in 20 ISS sessions within 2 years. Participant feedback showed a high effect and acceptance of training with reference to self-perceived impact and self-efficacy. Thirty-five team members experiencing 8 real critical events assessed team performance with TeamMonitor. Team performance assessment with TeamMonitor was feasible and identified specific areas to target future team training sessions. Training sessions as well as experienced real events revealed important latent safety threats that directed system changes. Conclusions: The programmatic approach of Kern's six steps for curriculum development helped to overcome barriers of design, implementation and assessment of an in-situ team and resuscitation training program. This approach may help improve effectiveness and impact of an ISS program. |
