# Supplementary material for: GENESISS 1—Generating Standards for In-Situ Simulation project: a scoping review and conceptual model
Source: BMC Med Educ. 2022 Jun 20;22:479. doi: 10.1186/s12909-022-03490-9 (PMC9208746; doi:10.1186/s12909-022-03490-9)
Supplement: Supplementary file 3 — Additional file 3. [file 12909_2022_3490_MOESM3_ESM.docx]

| Additional file 3 Stakeholder feedback | |
| --- | --- |
| *Who or what is the focus of investigation?* | ISS can be used to investigate the whole story of an incident, considering system structures, process and context. Incidents where clear responsibility has been attached to an individual or professional group or that involve simple isolated processes amenable to standardisation are less suitable for ISS investigations. Where incidents have had local consequences, ambiguity about ISS objectives could result in staff groups employing self-protective mechanisms and resisting full exploration of contributory factors. Participation should be voluntary and protect participant confidentiality. |
| *Is ISS already embedded within an open learning culture?* | Healthcare organisations where ISS is embedded as a method to improve safety and quality are best suited to implement ISS interventions which aim to understand and investigate clinical incidents. Conversely, ISS can exacerbate a ‘blame culture’ if the workplace is not an open and just learning environment. Whilst the aim is safe learning to improve care, facilitators should have pre-defined strategies to stop a scenario, offer time-out or switch to a more directive style where ‘unsafe’ practice is observed. |
| *What will ISS provide that a traditional investigation wouldn’t?* | ISS should complement rather than replace traditional systematic investigations of significant untoward incidents. If processes and mechanisms are influenced by the clinical environment or operate within dynamic systems, ISS may help illuminate HF components and interactions which result in different outcomes in particular circumstances. ISS should engage wide participation, encourage multidisciplinary team discussions, develop situational and self-awareness and enable staff to identify and implement their own solutions. |
| *What information and expertise do I need to plan scenarios and conduct ISS investigations?* | Simulation scenarios should be ‘based on’ actual incidents rather than aiming for exact replication. It may be more acceptable to design compound scenarios which are based on more than one incident, or incidents from other organisations, to promote systems thinking and protect the psychological safety of participants.  Multi-professional education and clinical teams should co-plan ISS scenarios, exploring the systemic factors involved in incidents and decide if and how these can be replicated and tested through ISS scenarios.  The accuracy of the source data used to plan scenarios should be considered. As with traditional approaches, ISS scenarios may introduce recall bias and the findings could be highly subjective. Although it may not be possible to overcome bias, assumptions can be challenged through multi-professional ISS involvement and reflexive accounts can help interpret the findings.  Skilled HF personnel are required to complete data collection and analysis which may involve expert observation, validated checklists and/or video capture. Field notes can capture timings, key action points and contextual factors. Pre/post measures of self-report confidence, knowledge and skills may also be helpful to evaluate the effectiveness of ISS. |
| *How would you feel if you were the staff member, patient or family member involved in the actual incident?* | Explore ways to involve staff and patients in the planning and conduct of ISS. Scenario plans should be presented to all stakeholder groups to gain their support prior to implementation. If there is potential to cause psychological harm to patients or staff, then ISS may not be appropriate. Appropriate resources and expertise should be available to conduct debrief sessions, provide support and help meet any training needs identified. |
| *How can I ensure the findings of ISS are useful?* | Findings should be developed into action plans and disseminated within and between healthcare organisations. Participants of ISS are encouraged to problem solve and have opportunity to experiment and explore solutions through subsequent ISS activities. Any urgent safety actions need to be reported to national and international patient safety organisations. It may be useful to compare the findings to actions points from official investigations or to current clinical guidelines, and highlight were improvements or clarifications are required. |
